# “Phage Transplantation in Allotransplantation”: Possible Treatment in Graft-Versus-Host Disease?

**DOI:** 10.3389/fimmu.2018.00941

**Published:** 2018-04-27

**Authors:** Andrzej Górski, Ewa Jończyk-Matysiak, Ryszard Międzybrodzki, Beata Weber-Dąbrowska, Jan Borysowski

**Affiliations:** ^1^Bacteriophage Laboratory, Hirszfeld Institute of Immunology and Experimental Therapy, Polish Academy of Sciences (HIIET PAS), Wrocław, Poland; ^2^Phage Therapy Unit, Hirszfeld Institute of Immunology and Experimental Therapy, Polish Academy of Sciences (HIIET PAS), Wrocław, Poland; ^3^Department of Clinical Immunology, Transplantation Institute, Medical University of Warsaw, Warsaw, Poland

**Keywords:** GvHD, hematopoietic cell transplantation, immunomodulation, inflammation, phage, phage therapy

## Abstract

Graft-versus-host disease, both acute and chronic (aGvHD, cGvHD) remains a major complication in patients undergoing hematopoietic cell transplantation (HCT) and a significant therapeutic challenge, as many patients do not respond adequately to presently available therapy. Increasing antimicrobial resistance has greatly revived interest in using bacterial viruses (phages) to combat antibiotic-resistant bacteria. In recent years, evidence has accumulated indicating that phages also have anti-inflammatory and immunomodulatory activities. This article suggests how these anti-bacterial and immunomodulatory activities of phages may be translated into a novel treatment of acute GvHD.

## Introduction

aGvHD is recognized as the most common serious complication after hematopoietic cell transplantation (HCT). It results from immune attack of T cells contained in the graft which recognize foreign tissues of the recipient, activating the lymphocytes, which subsequently mount an immune response against the grafted cells. New data also point to the role of microbial imbalance, the degree of which correlates with outcome and mortality. Typical aGvHD affects the skin (maculopapular rash), gastrointestinal tract (damage to epithelial cells), and liver (damage to small bile ducts). Recently, immunobiology and therapy of aGvHD have been discussed in detail (1, 2). While corticosteroids remain the mainstay of treatment, a substantial percentage of patients fail to respond (31% of standard risk patients and 57% of high-risk patients). Although several second-line therapies are available, their value still remains uncertain, so the outcome of such steroid-resistant patients is poor (2, 3). Evidently, novel treatment options are needed.

## New Insights into the Immunopathology of aGvHD—The Role of Neutrophils

### Experiments in Mice

Although T cells play a major role in the initiation of aGvHD recent data highlight the significance of neutrophils and their pro-inflammatory role in the pathogenesis of GvHD, especially its later stages. Schwab et al. (4) analyzed neutrophil infiltration of the mouse ileum following allogeneic HCT and found that physical or genetic depletion of neutrophils reduced the intensity and mortality related to GvHD. The number of granulocytes in cellular infiltrates of the intestinal tract is highly predictive of severe aGvHD and transplant-related mortality (5). Importantly, neutrophil-induced tissue damage in this setting required the production of reactive oxygen species (ROS): blocking ROS markedly reduced those effects. Neutrophil infiltration was dependent on commensal bacterial flora and toll-like receptor (TLR) activation in neutrophils (6).

### Clinical Data

In addition, the authors analyzed the number of neutrophils in patients after HCT and found that the severity of intestinal aGvHD strongly correlated with the number of neutrophils in aGvHD lesions and ROS-mediated oxidative injury. Furthermore, low rates of aGvHD were noted in patients with chronic granulomatous disease, whose neutrophils are deficient in ROS production (6). Granulocytes—generally considered as the first line of defense against pathogens—have recently been increasingly recognized also as antigen-presenting cells (APC) able to activate T lymphocytes. Activated T cells produce cytokines that upregulate major histocompatibility complex class II (MHC-II) and co-stimulatory molecule expression on neutrophils which differentiate into APC. Blocking of MHC-II on neutrophils abolishes their antigen-presenting function. In addition, neutrophils can activate themselves by interferon (IFN) gamma, which they produce. Upregulation of neutrophil MHC-II was shown in patients with active Wegener’s disease—neutrophils may present autoantigens and contribute to the development of autoimmune disorders; therefore, targeting neutrophils may be a new strategy to treat immune-mediated diseases (7).

## Microbiota and aGvHD

The importance of bacteria in the development of aGvHD was recognized decades ago. Initial studies showed the benefit of intestinal decontamination, but later studies failed to confirm those results—probably selective decontamination would be efficient (8). Loss of intestinal bacterial diversity is associated with the development of aGvHD; on the other hand, GvHD can lead to marked alterations of intestinal flora and expansion of Enterobacteriales (*Escherichia coli, Klebsiella, Enterobacter*, and *Enterococcus*). In another study, an increase in *Staphylococcaceae* was noted with an early onset of aGvHD, while a relative amount of >5% of *Enterobacteriaceae* was associated with increased mortality due to sepsis. Recent data indicate that the presence of oral *Actinobacteria* and *Firmicutes* in the stool was positively correlated with subsequent aGvHD (9–11). Narrow spectrum antibiotics and metronidazole reduce the risk of GvHD (9). These data suggest that microbiota manipulation can offer a new strategy of prevention and possibly treatment of aGvHD. Intervention in the gut microbiota using prebiotics and postbiotics may be a promising option (12).

Promising initial results were obtained using fecal microbiota transplantation (FMT) for the treatment of aGvHD (13). Fecal extracts lacking bacteria were effective in mediating beneficial effects of FMT, which may suggest that phages are responsible for those effects (14). In fact, successful FMT was associated with increased abundance of *Caudovirales* (15); thus, phages may be a key component of the microbiota responsible for the efficiency of FMT (16). Reduced phage richness was found in GvHD which is another argument for the role of phages in alleviating this pathology (17).

## Evolution of Phage Therapy (PT)

The dramatic increase of antimicrobial resistance and paucity of new antibiotics resulted in a growing interest in using bacterial viruses (phages) in combating antibiotic resistance (18, 19). Moreover, as pointed out, PT is evolving from treating complications to targeting diseases (20). While the safety of PT has been confirmed and clinical trials intended to provide full evidence of PT efficacy are underway, data have also accumulated indicating that PT mediates immunomodulating effects that could be useful in the clinic, especially in treating inflammatory and autoimmune disorders. As initially postulated, phages present in the human body (especially in the intestinal tract) could dampen exacerbated immune reactions and inflammatory responses and—by translocation from the gut to other tissues—contribute to maintenance of immune homeostasis at the level of both the intestines and other tissues (“natural phage therapy”) (21, 22). This hypothesis was confirmed by Barr (23), who demonstrated that phages adhering to mucus protect the underlying epithelium from bacterial infection. Recently, we suggested that in fact phages could not only protect gut epithelium from bacterial invasion but also interact with the epithelial cells to directly inhibit inflammation (e.g., through dampening ROS production by those cells and/or infiltrating neutrophils) (24). Previously, we have shown that homologous (but not heterologous) phages inhibit ROS production in human neutrophils and monocytes induced by *E. coli*; phages themselves induce only minimal ROS production in phagocytes (25). This phenomenon was later confirmed and extended, revealing that phages inhibit ROS induced in neutrophils not only by bacteria but also by endotoxin (26), while the inability of T4 phage and its capsid proteins to induce ROS has been confirmed by Miernikiewicz et al. (27). Importantly, phages do not induce neutrophil degranulation (28). Phages and some of their capsid proteins have also been demonstrated to inhibit inflammatory skin and organ infiltrates in experimental mice, skin allograft rejection in mice, and autoimmune reaction using a mouse model of rheumatoid arthritis (29–31). Recently, Van Belleghem et al. demonstrated a predominantly anti-inflammatory effect of phages as evaluated by their ability to influence expression and production of cytokines and specific receptors of human mononuclear cells (32). This correlates with laboratory data derived from patients on PT indicating that the therapy significantly dampens C-reactive protein (CRP) and the sedimentation rate in patients (33); in fact, in some patients, a reduction in CRP may occur shortly after the onset of therapy even though eradication of infection has not been achieved (20). Moreover, reduction of proteinuria may take place in those patients (34); as proteinuria may be considered another marker of inflammation, this finding provides another argument for anti-inflammatory action of phages (35, 36).

We postulated that intestinal phages can translocate from the gut into the blood and other organs and tissues, mediating immunomodulatory activities which may be relevant for maintenance of immune homeostasis (“natural phage therapy”) (21, 22). These assumptions have recently been confirmed by other groups. It has been demonstrated that phages are transcytosed across epithelial cell layers (>30 billion phages daily). In line with those observations, Lehti et al. (37) have shown that phages can bind and penetrate into neuroblastoma cells *in vitro* and persist inside human cells up to 1 day without phage entry into the nucleus and without affecting cell viability. Those data strongly suggest that “natural phage therapy” (encompassing not only anti-bacterial activities of phages but also their anti-inflammatory and immunomodulating functions) may be translated into novel forms of clinical PT reaching well beyond current antimicrobial applications.

## Potential of Phages to Mitigate the Severity of an Experimentally-Induced and Clinical aGvHD

As pointed out, ROS production by neutrophils is an important hallmark of GvHD. In this context, the ability of phages to dampen such ROS production provides a strong argument for their potential use in this setting, especially as PT does not impair human granulocyte and monocyte ability to kill standard strains and pathogens isolated from patients—thus, the therapy should not contribute to immunodeficiency in patients undergoing HCT. In fact, PT does not induce immunodeficiency and may be used in immunodeficient patients (38, 39). It should be noted that the reduction of oxidative stress may be beneficial in mice with aGvHD (40, 41). Furthermore, neutrophils can also exert beneficial effects in aGvHD when primed with G-CSF. Such neutrophils have diminished expression of MHC-II and co-stimulatory molecules, decreased IFN gamma production but high IL-10 production, consistent with “suppressor neutrophils” inducing donor Tregs responsible for aGvHD suppression in mice (42). The significant beneficial role of IL-10 in aGvHD is supported by data derived from cord blood (CD) transplantation and clinical observations indicating that CD recipients have lower incidence of aGvHD. CD contains an abundance of IL-10 producing B cells which suppress T cell proliferation and effector functions with the aid of that cytokine. In addition, there was a marked recovery of IL-10 producing B cells to levels found in healthy donors (43). These findings are supported by the data derived from patients following HCT in whom polymorphism in the IL-10 promoter region was found to have a significant effect on the outcome of transplantation. The authors believe that a high level of IL-10 production by the recipient’s cells during the early post-transplant period mitigates the alloantigen-induced immune response and GvHD-induced inflammation (44). In line with those observations, Chan et al. demonstrated that NK cells rapidly reconstitute following clinical HCT and produce IL-10, which may suppress alloimmune T cell-mediated responses (45). In a mouse model of aGvHD, IL-10 gene-modified dendritic cells induced transplant tolerance and suppressed aGvHD (46). IL-10 has also been shown to downregulate TLR-mediated human dendritic cell activation (47). In this context, the recent data of Van Belleghem et al. indicating that phage induce IL-10 production in human mononuclear cells appear to be of paramount importance (32). Previously, it has been reported that indeed phage preparations may induce IL-10 in those cells (38); in addition, phage films may induce IL-10, reducing inflammatory responses (48). However, it should also be kept in mind that IL-10 has been shown to support the growth of cytotoxic T cells and clinical studies in patients with Crohn’s disease failed to show its efficacy (49).

As already mentioned, TLR activation enhances neutrophil infiltration of GvHD lesions. Most studies confirm the role of lipopolysaccharide (LPS) and TLR4 in the development of aGvHD. In experimental bone marrow transplantation in mice, LPS antagonism reduces aGvHD while preserving graft-versus-leukemia activity (50). TLR knockout mice have attenuated aGvHD (51). Heparan sulfate, a TLR4 agonist, promotes aGvHD following bone marrow transplantation in mice (52). Although there are also data suggesting that in some experimental models TLR4 signaling may not be not absolutely required for the development of GvHD, the existing data suggest that targeting LPS and TLR4 signaling is a promising area to search for new GvHD treatment modalities (53). Phages may interfere with LPS and reduce its pro-inflammatory effects (including reduction of organ and tissue infiltration) (54) and dampen TLR expression (32).

Blockade of IL-1 reduces inflammation, and therefore this area of research is of obvious interest in studies on novel GvHD treatment modalities. IL-1 blockade indeed reduces aGvHD development, while IL-1 receptor deficiency alleviates murine aGvHD (55, 56). The donor genotype for the IL-1 receptor antagonist (IL-1Ra) polymorphism has a clear protective role against aGvHD in the clinic (57). While a clinical trial involving IL-1Ra did not show its effectiveness in preventing aGvHD, the authors believe that the agent was discontinued too early or different schedules or dosing would give different results (58). Moreover, the level of IL-1Ra was found to be markedly reduced in saliva from patients with cGvHD (59), which highlights IL-1Ra as a promising agent for future studies on its potential beneficial effect not only in aGvHD but also cGvHD. In this context, it should be noted that phages increase the production of this cytokine in human mononuclear cells (32).

Azithromycin (AZM) is active against a variety of bacteria and is also an anti-inflammatory agent modulating the functions of dendritic cells, monocytes, and granulocytes, inhibiting the NF-kappaB pathway following LPS stimulation. It also stimulates production of IL-10 *in vitro*. In a mouse model of aGvHD, a short course of AZM suppressed aGvHD significantly, whereas lymphocyte functions were unchanged. The authors suggest that AZM may be a novel prophylactic agent for lethal GvHD (60). Interestingly, phages exhibit similar activities to AZM: they may eliminate bacteria (in contrast to AZM phages they act very selectively, so specific bacterial targeting allowing for elimination of a given species of bacterium is possible); in addition—as pointed out earlier—phages have immunomodulating and anti-inflammatory effects (including inhibition of NF-kappaB) (54). Therefore, phages may reduce skin, gut, and liver infiltration typical for aGvHD, inhibit ROS production important for immunopathology of aGvHD, and induce IL-10, alleviating this condition. Gastrointestinal manifestations of aGvHD are often severe and pose a significant therapeutic challenge, while immune-mediated damage to intestinal epithelial cells (IEC) is a hallmark of this condition (2). We postulate that phages may interact with IEC, protecting those cells from such damage, which could offer a new form of immunotherapy of inflammatory bowel diseases (IBD) (24). There are many similarities between IBD and aGvHD (61), so this concept might also be applicable in treating aGvHD.

## Could PT be Potentially Beneficial in cGvHD as Well?

The immunopathologic mechanisms responsible for cGvHD are activated already at the time of HCT and include inflammation, cell-mediated and humoral immunity, aberrant immunoregulation, and fibrosis (62). Clinical manifestations of cGvHD are similar to autoimmune collagen diseases (63).

There is no specific prophylactic therapy of cGvHD. Corticosteroids remain the mainstay of therapy for established syndrome with immunosuppressants as second-line therapy. However, current regimens have limited efficacy and significant side effects; about one-third of patients treated at the best centers relapse or die (64). In this regard, it is interesting that our group has recently reported that PT may be beneficial in a mouse model of collagen-induced autoimmune syndrome (31). A review of other available data on anti-inflammatory and immunomodulating effects of phages presented in this article (for example, low levels of IL-1Ra in cGvHD and upregulation of that cytokine by phages) suggests that PT could also bring benefits to patients with cGvHD (perhaps as an adjunct therapy allowing for steroid sparing).

## Conclusion

The perspective presented herein is speculative, but may stimulate new directions of research that could result in a significant progress in transplantation. To obtain some preliminary data, a clinical trial of PT in IBD would be prudent before initiating such trials in immunosuppressed patients with aGvHD. Selection of most appropriate phage(s) should be based on available information on its immunomodulating activities that could be instrumental in alleviating the immunopathology of GvHD. As stated earlier, data supporting the potential applicability of T4 phages has been supplied by our group, while the data of Van Belleghem et al. (32) also suggest the potential of *Staphylococcus aureus* and *Pseudomonas* phages. It may well be that the optimal immunomodulatory effects could be mediated by a cocktail of phages targeting different immune cell receptors and cytokines. Such monovalent or polyvalent phage preparations could be produced using standard technology currently used for production of anti-bacterial phage preparations (65, 66).

aGvHD usually occurs within 100 days after HCT; therefore, a clinical trial should cover that initial period following transplantation as aGvHD prophylaxis. Such a trial should involve patients who received HCT for correction of hemopoietic deficiency (e.g., aplastic anemia) rather than patients with leukemia (to avoid serious complications like recurrence of malignancy).

aGvHD prophylaxis with cyclosporine is used in the majority of patients. Despite this prophylaxis, aGvHD may still evolve (1). Therefore, in addition to cyclosporine, phages could be added. Oral phage administration would be preferred as the intestinal tract is the common site of GvHD-related immunopathology; furthermore, such administration usually does not induce significant anti-phage antibody production while phages can penetrate the gut barrier and act also at other tissue sites (22, 23). Detailed description of PT protocol has been provided earlier (34).

Viruses provide balance to the holobiont, keeping the host and associated prokaryotes and eukaryotes functioning together as a unit (67). If indeed our endogenous phages—referred to as “bacteriophage guests”—protect human health (68), then PT using exogenous phages (which may be considered as a form of transplantation) should produce similar beneficial effects. PT in HCT may help combat antibiotic-resistant infections in those patients, enable selective decontamination and therapy-oriented manipulation of the gut microbiome, and attenuate inflammation and aberrant immunity. Therefore, PT offers new tools for personalized, preemptive, and therapeutic strategies to improve HCT results, which should be explored further.

## Author Contributions

AG drafted the main part of the manuscript; EJ-M, RM, BW-D, and JB contributed parts of the manuscript; all authors approved the manuscript.

## Conflict of Interest Statement

AG, RM, BW-D, and JB are co-inventors of patents owned by the Institute of Immunology and Experimental Therapy, Wrocław, covering phage preparations. The remaining author declares that they have no conflicts of interest.
